# A Sigh of Relief

**Published:** 1892-10

**Authors:** 


A SIGH OF RELIEF.—It is amusing to see how little will sometimes make a weak, conceited man lose his temper and make a spectacle of himself. Last month we said, "The Happy Medium; we are glad to know it is happy, it is certainly medium."

Whereupon the "Happy Medium" man got unhappy, and called us "Red Eyed Bill" (or Dick) from some sort of a "gulch."

That's not medium; that's coarse.

He also calls the JOURNAL "Daniel's Texas *Steer*." That, we suppose is wit; a loose kind of wit—the *Bransford* Lewis kind. He says, too, that he cannot elevate us to the level of his contempt. Too bad. (When he wanted a little free advertising he called on our "esteemed journal.")

That standing conundrum, "A popular querry, what will the *Fortnightly* say about it?" is now answered; the *Fortnightly* has said about it, and the country breathes freer. *That* agony and the cholera both over at once! Goody-goody, glad!

Now let everybody take one of *Fortnightly's* "shot gun prescriptions," "original communications, grains 2; book notices, grains 2," etc., (some more wit) and be happy. Providence was kind in bestowing a Bransford Lewis on journalism; what did we ever do without him?

